# Conservation of structural brain connectivity in people with multiple sclerosis

**DOI:** 10.1162/netn_a_00404

**Published:** 2024-12-10

**Authors:** Gerard Martí-Juan, Jaume Sastre-Garriga, Angela Vidal-Jordana, Sara Llufriu, Eloy Martinez-Heras, Sergiu Groppa, Gabriel González-Escamilla, Maria A. Rocca, Massimo Filippi, Einar A. Høgestøl, Hanne F. Harbo, Michael A. Foster, Sara Collorone, Ahmed T. Toosy, Menno M. Schoonheim, Eva Strijbis, Giuseppe Pontillo, Maria Petracca, Gustavo Deco, Àlex Rovira, Deborah Pareto

**Affiliations:** Neuroradiology Group, Vall d’Hebron Research Institute (VHIR), Barcelona, Spain; Department of Neurology/Neuroimmunology, Multiple Sclerosis Centre of Catalonia (Cemcat), Vall d’Hebron University Hospital, Universitat Autònoma de Barcelona (UAB), Barcelona, Spain; Neuroimmunology and Multiple Sclerosis Unit and Laboratory of Advanced Imaging in Neuroimmunological Diseases (ImaginEM), Hospital Clinic Barcelona, Fundació de Recerca Clínic Barcelona-IDIBAPS and Universitat de Barcelona, Barcelona, Spain; Department of Neurology, Focus Program Translational Neuroscience (FTN) and Immunotherapy (FZI), University Medical Centre of the Johannes Gutenberg University Mainz, Rhine Main Neuroscience Network (rmn2), Mainz, Germany; Neuroimaging Research Unit, Division of Neuroscience, IRCCS San Raffaele Scientific Institute, Milan, Italy; Neurology Unit, IRCCS San Raffaele Scientific Institute, Milan, Italy; Vita-Salute, San Raffaele University, Milan, Italy; Department of Neurology, Oslo University Hospital, Oslo, Norway; Department of Psychology, University of Oslo, Oslo, Norway; Institute of Clinical Medicine, University of Oslo, Oslo, Norway; Queen Square MS Centre, Department of Neuroinflammation, Queen Square UCL Institute of Neurology, University College London, London, United Kingdom; Anatomy and Neurosciences, MS Centre Amsterdam, Amsterdam Neuroscience, Amsterdam UMC, Vrije Universiteit Amsterdam, Amsterdam, Netherlands; Neurology, MS Center Amsterdam, Amsterdam Neuroscience, Amsterdam UMC, Vrije Universiteit Amsterdam, Amsterdam, Netherlands; Departments of Advanced Biomedical Sciences and Electrical Engineering and Information Technology, University of Naples “Federico II”, Naples, Italy; Department of Neurosciences and Reproductive and Odontostomatological Sciences, University of Naples “Federico II”, Naples, Italy; Department of Human Neurosciences, Sapienza University of Rome, Naples, Italy; Centre for Brain and Cognition, Computational Neuroscience Group, Department of Information and Communication Technologies, Universitat Pompeu Fabra, Barcelona, Spain; Institució Catalana de la Recerca i Estudis Avançats, Universitat Pompeu Fabra, Barcelona, Spain; Neuroradiology Section, Radiology Department (IDI), Vall d’Hebron University Hospital, Barcelona, Spain

**Keywords:** Multiple sclerosis, Hemispheric connectivity, MRI, Structural connectivity

## Abstract

Multiple sclerosis (MS) is a neurodegenerative disease that affects the central nervous system. Structures affected in MS include the corpus callosum, connecting the hemispheres. Studies have shown that in mammalian brains, structural connectivity is organized according to a conservation principle, an inverse relationship between intra- and interhemispheric connectivity. The aim of this study was to replicate this conservation principle in subjects with MS and to explore how the disease interacts with it. A multicentric dataset has been analyzed including 513 people with MS and 208 healthy controls from seven different centers. Structural connectivity was quantified through various connectivity measures, and graph analysis was used to study the behavior of intra- and interhemispheric connectivity. The association between the intra- and the interhemispheric connectivity showed a similar strength for healthy controls (*r* = 0.38, *p* < 0.001) and people with MS (*r* = 0.35, *p* < 0.001). Intrahemispheric connectivity was associated with white matter fraction (*r* = 0.48, *p* < 0.0001), lesion volume (*r* = −0.44, *p* < 0.0001), and the Symbol Digit Modalities Test (*r* = 0.25, *p* < 0.0001). Results show that this conservation principle seems to hold for people with MS. These findings support the hypothesis that interhemispheric connectivity decreases at higher cognitive decline and disability levels, while intrahemispheric connectivity increases to maintain the balance.

## Author Summary

In our study, we investigated how multiple sclerosis (MS), a disease that affects the central nervous system, impacts brain connectivity and the conservation principle of connectivity in the brain across hemispheres. By analyzing data from 513 MS patients and 208 healthy individuals, we examined if this conservation principle holds and how it changes due to MS. Our findings revealed that both MS patients and healthy individuals exhibit a similar balance between connections within each hemisphere and between hemispheres. We also observed that as cognitive impairment and disability in MS patients increase, interhemispheric connectivity decreases while intrahemispheric connectivity compensates. This suggests that the brain attempts to maintain balance despite the disease’s progression, highlighting the adaptability of neural connectivity during the course of MS.

## INTRODUCTION

Multiple sclerosis (MS) is a disabling disease that affects the central nervous system (Thompson et al., 2018). People with MS (pwMS) exhibit a wide spectrum of disease presentations with varying symptoms, including visual impairment, motor disability, and cognitive decline. Atrophy (Sastre-Garriga et al., 2020), focal and diffuse white matter damage (Kutzelnigg et al., 2005), and brain functional connectivity alterations (d’Ambrosio et al., 2020; Rocca et al., 2018) can be present in the central nervous system of pwMS, captured with both conventional and advanced MRI methods.

Regarding brain connectivity, MS has been associated with disruptions in structural connectivity (SC) (Fleischer et al., 2019). Associations have been found between white matter lesion load and structural network disconnection (He et al., 2009; Llufriu et al., 2017; Pagani et al., 2020). When defining the SC as a connected graph, measures like network efficiency were found to be associated with disability and disease duration (Shu et al., 2011, 2016). More specifically, atrophy in the corpus callosum (CC) (Ozturk et al., 2010; Sigal et al., 2012) and thalamus (Schoonheim, Hulst, et al., 2015), structures that connect both brain hemispheres, have been linked to a decline in cognition and an increase in disability for pwMS. Interhemispheric connections are needed to maintain small-worldness and network efficiency (Watts & Strogatz, 1998), so those disruptions have the potential to alter the normal patterns of brain connectivity.

Given these disruptions on these key structures, it is of interest to explore the brain connectivity in MS and how it could relate to the observed alterations. The mammalian brain has been shown to present a conservation principle of structural brain connectivity across species and different brain structures (Assaf et al., 2020), with intrahemispheric brain connectivity increasing as interhemispheric brain decreases, and vice versa. This association between intra- and interhemispheric connectivity has also been observed in humans. Krupnik et al. (2021) showed that this relationship holds in the human brain by analyzing a cohort of 1,497 subjects and their SC and suggesting that a lower interhemispheric connectivity was associated with lower crystallized intelligence. This conservation of SC, however, has not been studied in MS. Zhou et al. (2013) tested if interhemispheric homotopic connections were affected in MS studying functional MRI and the CC, finding reduced interhemispheric connections in pwMS. Petracca et al. (2020) showed reduction in CC streamline density related to white matter damage. Those works, however, did not assess the behavior of the intrahemispheric connectivity. Given these disruptions caused by MS on SC, we believe that it is relevant to investigate whether such conservation is affected by the disease and any possible connection to physical and cognitive disability.

In this work, we analyzed the brain connectivity of 514 pwMS and 216 healthy controls (HC) from seven different centers, using SC to study the association between intrahemispheric and interhemispheric connectivity in MS. The primary aim of this study was to analyze if the conservation of brain SC holds for pwMS, and how it relates to the physical and cognitive disability caused by MS. Our initial hypothesis was, given the available evidence (Assaf et al., 2020; Krupnik et al., 2021), that the conservation will hold, but a lower interhemispheric connectivity caused by MS (Ozturk et al., 2010; Schoonheim, Hulst, et al., 2015; Sigal et al., 2012) will be observed, which will be compensated by an increase of intrahemispheric connectivity. We extracted different intra- and interhemispheric connectivity markers for each subject, studied their interactions, and showed their associations with cognition and disability for pwMS.

## MATERIALS AND METHODS

### Data

Data for this project were provided by members of the European Magnetic Resonance Imaging in MS (MAGNIMS) consortium. Seven centers have participated in the study, in no specific order: Hospital Clínic, Institut Investigació Biomèdica August Pi i Sunyer, Barcelona, Spain; University Medical Centre of the Johannes Gutenberg, Mainz, Germany; Istituto di Ricovero e Cura a Carattere Scientifico Ospedale San Raffaele, Milan, Italy; Università degli Studi di Napoli “Federico II,” Naples, Italy; Oslo University Hospital, Oslo, Norway; Amsterdam UMC, Amsterdam, Netherlands; and University College London, London, United Kingdom.

Participants were recruited at each center and data were transferred within a MAGNIMS general framework agreement. The study participants underwent MRI scans using 3T scanners, which provided both conventional (T1 and Fluid attenuated inversion recovery) and advanced Diffusion-weighted imaging (DWI) imaging. Physical (Expanded Disability Status Scale, EDSS) and cognitive (Symbol Digit Modality Test, SDMT) outcomes were also provided. EDSS captures the overall disability in pwMS, while SDMT is a cognitive function measure that captures information processing speed, attention, and visual model coordination. PwMS were divided between low/high EDSS (cutoff value of 3, as in Leray et al., 2013) and low/high SDMT (cutoff value of 40, as described in Van Schependom et al., 2014, for differentiating cognitive impairment) for later analysis. Information about the specific imaging protocols provided by each center is available in Supporting Information Data S1.

After data processing and quality control (see the Data Processing section), the final cohort contained a total of 697 subjects. Table 1 shows the age and sex (and for pwMS, disease duration, EDSS, and SDMT) of the subjects, divided by center. Further information and distribution of values (brain parenchymal fraction, EDSS, SDMT, and lesion volume fraction [LVF]) across centers can be found in Supporting Information Figure S2.

**Table 1.** Cohort information

	Amsterdam	Barcelona	London	Mainz	Milan	Naples	Oslo
*N* - HC	48	8	19	26	30	53	24
*N* - pwMS	173	58	43	50	56	51	58
*N* - total	221	66	62	76	86	104	82
Age - HC	48.41 ± 9.3	29.94 ± 10.6	33.19 ± 7.0	27.85 ± 6.4	37.26 ± 9.3	41.30 ± 11.6	35.12 ± 8.7
Age - pwMS	48.80 ± 11.3	48.81 ± 9.6	34.43 ± 7.9	35.78 ± 11.6	42.18 ± 9.7	42.48 ± 12.9	40.59 ± 7.2
Sex - HC	58.33%	87.50%	63.16%	50.00%	40.00%	62.26%	62.50%
Sex - pwMS	71.68%	72.41%	62.79%	64.00%	55.36%	66.67%	70.69%
EDSS	3.5 (2.5, 5.5)	2.5 (1.5, 3.875)	1.5 (1.0, 2.0)	1.5 (1.0, 2.0)	3.75 (1.5, 6.125)	4.5 (2.5, 6.0)	2.0 (1.5, 2.875)
SDMT	51.21 ± 13.3	46.05 ± 13.4	58.84 ± 9.9	53.12 ± 11.3	50.95 ± 14.3	41.29 ± 13.8	51.48 ± 9.5
DD	15.26 ± 8.7	19.54 ± 9.4	0.41 ± 0.5	4.99 ± 6.6	10.81 ± 9.8	13.29 ± 9.0	10.09 ± 5.3

Age (years), EDSS is shown in the format median (25%–75% IQR). SDMT is shown in the format mean ± *SD*. Sex is shown as the percentage of females over the total. Abbreviations; HC = healthy controls; pwMS = people with MS; EDSS = Expanded Disability Status Scale; SDMT = Symbol Digit Modality Test; DD = disease duration (years).

### Data Processing

All the data were processed using with a single machine (Intel Xeon with 24 cores at 3.50 GHz, 128-GB RAM, NVIDIA Quadro RTX 5000 GPU). Subjects were processed in parallel when possible. The code for MRI preprocessing pipeline, including structural processing, lesion segmentation, diffusion preprocessing, and fiber tracking, is available at https://github.com/GerardMJuan/FC-SC-data-pipeline.

#### Structural preprocessing.

The three-dimensional T1 was segmented and parcellated in the Desikan-Killiany atlas with FastSurfer (Henschel et al., 2020, 2022). The final segmentation included 60 cortical and 16 subcortical regions. Gray matter fraction (GMF) and white matter fraction (WMF), compared with intracranial volume were also computed for all subjects for later analysis.

#### Lesion segmentation.

Hyperintense white matter lesions in pwMS were segmented using the Lesion Segmentation Toolbox (Pareto et al., 2016; Schmidt et al., 2012), using the default lesion growth algorithm with the registered T1 and FLAIR. We also computed the LVF for all pwMS.

#### Diffusion preprocessing.

Diffusion image processing was performed using MRtrix3 (Tournier et al., 2019). We performed denoising, correction of Gibbs ringing (Kellner et al., 2016), and distortion correction (S. M. Smith et al., 2004), which included correction for eddy current-induced distortion, motion correction, and, if possible, when data were available, fieldmap-based unwarping using PRELUDE (Jenkinson, 2003) or inhomogeneity distortion correction using TOPUP (Andersson & Sotiropoulos, 2016) and bias correction. This step was skipped for the centers where no extra volumes with reversed gradient polarity for correction were available.

Fiber tracking was conducted using a single shell or multishell (depending on the data characteristics) based on the constrained spherical deconvolution method (iFOD2 algorithm [Tournier et al., 2010]) to estimate the fiber orientation distributions (Jeurissen et al., 2014; Tournier et al., 2007), using Dhollander’s algorithm (Dhollander et al., 2019) to estimate the response function. This was accomplished by utilizing the available segmentation of tissues (gray matter, white matter, and cerebrospinal fluid) created during the structural preprocessing, registering them to the diffusion scan, as well as white matter lesion segmentation, to establish an anatomically constrained tractography (ACT) (R. E. Smith et al., 2012). Finally, 6,000,000 streamlines were generated using *tckgen* from MRtrix3 (Tournier et al., 2019), using seeding at random inside the brain, with the ACT framework (R. E. Smith et al., 2012), allowing backtracking during tracking if a poor structural termination is encountered, with a cutoff value of 0.06, cropping streamline endpoints as they cross the GM-WM interface.

To reduce the number of biologically unrealistic streamlines in the generated tractography, an automatic anatomical exclusion criteria algorithm was employed (Martínez-Heras et al., 2015). We extend the implementation described in the paper to every pair of regions from our previous segmentation to remove the implausible streamline between each pair of regions. Then, the SIFT2 algorithm (R. E. Smith et al., 2015) was used to assign a weight to each streamline based on its likelihood of being biologically plausible, allowing to use the streamline count as a biological marker of connection, as well as allowing quantitative comparison across subjects (R. E. Smith et al., 2022). The final streamline to node assignment was done using a radial search, with the threshold being the slice thickness of the scan plus 0.5. Finally, the resulting SC matrix was divided by fiber length. This last step was done to further remove bias toward longer fibers (Hagmann et al., 2008; Roberts et al., 2016) and to incorporate fiber length across nodes to our connectivity analysis, given its relevancy to brain connectivity (Bajada et al., 2019).

#### Quality control.

Quality control was performed by manually checking the segmentation of the cortical and subcortical regions, the lesion segmentation, and the registration of structural and diffusion scans. Any subjects that showed poor registrations (bad alignment) across sequences, inferior quality scans (noisy scans, low contrast across tissues), or incorrect segmentations were removed. This was facilitated by an automated script, included in the repository.

### Hemispheric Connectivity Measures

A total of 76 cortical and subcortical areas, including the CC, were segmented across the brain, as described in the previous section, and the scans and segmentations were registered to a common template. The SC matrices were derived from fiber tracking across 76 cortical and subcortical regions using the same parcellation scheme (76 nodes). For computing the intra- and interhemispheric connectivity measures, we considered only connections between cortical areas, as in Assaf et al. (2020) and Krupnik et al. (2021). We were left with 60 cortical regions, 30 per hemisphere. The weights of the connectivity matrix were created by doing the inverse of the connections.

#### Interhemispheric connectivity.

Interhemispheric connectivity was evaluated using two different metrics, as in Assaf et al. (2020): the commissural ratio and the CC ratio:

$$\text{commissural}\;\text{ratio}=\frac{\text{N}{}^{\circ}\;\text{streamlines}\;\text{crossing}\;\text{hemispheres}}{\text{Total}\;\text{N}{}^{\circ}\;\text{streamlines}}$$ (1)

$$\text{CC}\;\text{ratio}=\frac{\sqrt{CC_{\text{area}}}}{\sqrt[{3}]{\text{Brain}\;\text{Vol}.}}$$ (2)

where *CC*_area_ is the midsagittal area of the CC, and BrainVol. is the total brain volume. For the commissural ratio, the number of streamlines crossing hemispheres was computed as the total number of streamlines where the start and the end were in opposed hemispheres (Assaf et al., 2020). The commissural ratio is computed from the fiber tracking, while the CC ratio comes only from volumetric data derived from T1 structural imaging. Both were used and compared to evaluate how well they were related and how they captured interhemispheric connectivity.

#### Intrahemispheric connectivity.

As with the interhemispheric connectivity analysis, two different measures were computed.

- **Shortest path length (SPL)**: The mean SPL of a graph is a measure of the efficiency of information flow in the graph. It is defined as the mean of the SPL between all pairs of nodes. Mathematically, it can be defined as follows:
  $$\frac{\sum_{i=1}^{N}\sum_{j=i+1}^{N}d\left(i,j\right)}{N\left(N-1\right)}$$ (3)
  where *d*(*i*, *j*) is the SPL between nodes *i* and *j*, and *N* is the number of nodes in the graph.
- **Efficiency**: The efficiency of a network quantifies the extent of the network integration, that is, a measure of the overall capacity for information transferring across nodes or regions in the brain (Bullmore & Sporns, 2012). Efficiency is defined as the mean of the inverse of the SPL across all nodes in the graph. Mathematically, it can be defined as follows:
  $$\frac{\sum_{i=1}^{N}\sum_{j=i+1}^{N}\frac{1}{d\left(i,j\right)}}{N\left(N-1\right)}$$ (4)
  where *d*(*i*, *j*) is the SPL between nodes *i* and *j*, and *N* is the number of nodes in the graph.

The mean SPL and efficiency were calculated separately for each hemisphere and averaged. Those measures were used to (a) replicate the results observed in Assaf et al. (2020) and Krupnik et al. (2021) and (b) observe any differences between HC and pwMS on intra- and interhemispheric connectivity.

Both the mean SPL and the efficiency measure information flow in networks, and they mostly act as the inverse of the other. However, they offer distinct interpretations and sensitivities. Efficiency is more robust in networks with disconnected nodes or infinite path lengths, as it accounts for the inverse of path lengths, while SPL is more sensitive to changes in the longest paths within the network (Rubinov & Sporns, 2010). Because of this, and with both being used in the two papers we want to replicate (Assaf et al., 2020; Krupnik et al., 2021), we have decided to include both in our analysis.

### ComBat Harmonization

ComBat harmonization was employed to correct for substantial intersite variability across the seven centers involved in data acquisition. ComBat harmonization (Fortin et al., 2018; Johnson et al., 2007) is a statistical technique designed to remove site-specific variability in multicenter neuroimaging studies, thereby enhancing the comparability of data collected across different scanners and protocols. This method utilizes empirical Bayes frameworks to adjust for scanner-related effects while preserving biological variability and effects of interest. This approach is important in reducing the confounding influence of site-specific differences, facilitating more accurate comparisons and interpretations of the data.

As described in the Data section, data from seven different centers, exhibiting large differences in scanner models, acquisitions parameters, and protocols, were included (Supporting Information Data S1 contains the full details of the differences in acquisition parameters across centers). ComBat harmonization was applied using the Python implementation provided by the authors of the paper (https://github.com/Jfortin1/neuroCombat). Age, sex, and diagnosis were set as covariates. Diagnosis was selected as a proxy for EDSS/SDMT, as we did not have those values available for controls. The correction was applied to all neuroimaging-derived values from MRI, diffusion, and hemispheric connectivity measures. The corrected values were then used for all subsequent experiments that were not separated by center.

### Statistical Analysis

All statistical analysis experiments were implemented in Python using the statsmodels package. The analysis of brain networks was implemented in Python using the networkX package.

The relationship between intra- and interhemispheric connectivity was assessed by computing the correlation between pairs of inter-/intrahemispheric connectivity values, divided between HC and pwMS, regressing out age and sex. The same analysis was done for each center separately, to check if the relationship also holds. Regarding interhemispheric connectivity, the correlation between the two measures (commissural ratio and CC ratio) was assessed to analyze how the two variables were related.

Correlations were run between inter- and intrahemispheric connectivity values and GMF, WMF, LVF, and disability and cognitive scores (regressing out age, sex, and center). Prior to analysis, we checked if SDMT was correlated with the years of education in pwMS. After correcting for age and sex, we did not observe significant associations between education and SDMT, so it was not included in the model (Supporting Information Table S3). Differences in intra- and interhemispheric values across HC and pwMS divided in high and low EDSS and SDMT groups were computed using analysis of variance (ANOVA) with three groups, and differences across groups were evaluated with post hoc pairwise Tukey honestly significant difference (HSD) tests.

To further analyze how the relationship between intra- and interhemispheric SC varies in MS, we also evaluated how the ratio (inter/intra) of the values (four ratios in total) relates to EDSS and SDMT. We repeated the same experiments as before with the calculated ratios. All tests described in this section were corrected for multiple comparisons using Bonferroni correction.

## RESULTS

**Figure 1.**
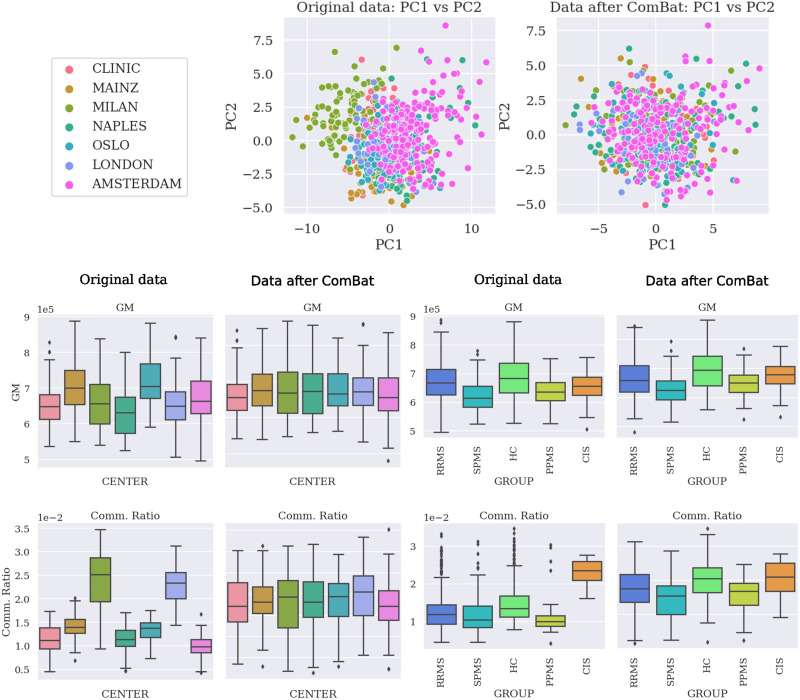
Comparison of the data before and after applying the ComBat harmonization procedure. Colored by center. Top: Comparison between the two top principal components after a PCA. Bottom: Distribution separated by center (left) and group (right) before and after ComBat harmonization. PCA = principal components analysis; GM = gray matter; RRMS = relapsing-remitting MS; SPMS = secondary progressive MS; HC = healthy control; PPMS = primary progressive MS; CIS = clinically isolated syndrome.

**Figure 2.**
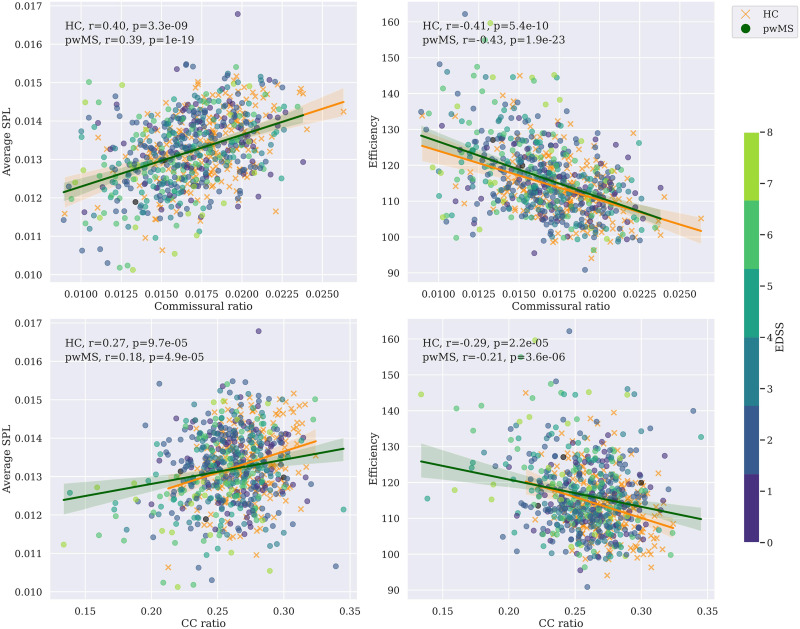
Correlation between pairs of intra/interhemispheric SC measures. Each plot shows the Pearson correlation between the two variables, with the *x*-axis representing the interhemispheric value and the *y*-axis representing the intrahemispheric value. pwMS are colored by EDSS. HC is marked with a cross. Values are corrected by age and sex. Data are harmonized across centers using ComBat. Tests results are corrected for multiple comparisons. EDSS = Expanded Disability Status Scale; HC = healthy controls; pwMS = people with MS; SPL = shortest path length; CC = corpus callosum.

**Figure 3.**
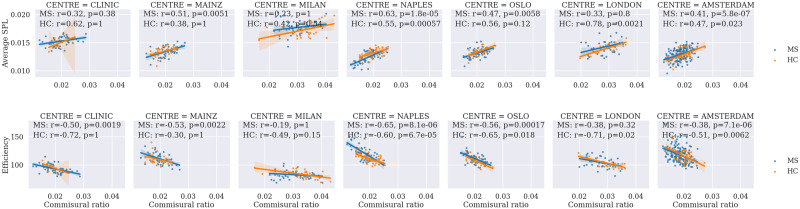
Correlation between commissural ratio and SPL/efficiency SC measures, separated by center. Each plot shows the Pearson correlation between the two variables, with the *x*-axis representing the interhemispheric value and the *y*-axis representing the intrahemispheric value. pwMS is colored blue, and HC is colored orange. Values are corrected by age and sex. Results are corrected for multiple comparisons. MS = multiple sclerosis; HC = healthy controls; pwMS = people with MS; SPL = shortest path length.

Thirty-tree subjects were removed from the cohort after quality control, leaving the total number of subjects at 697, described in the Data section. Results of combat harmonization are shown in Figure 1, comparing the distribution of two specific imaging markers (GM and commissural ratio), as well as the principal components analysis (PCA) of all the imaging markers, to visualize the changes after harmonization. Supporting Information Figure S4 shows more differences across other imaging markers before and after Figure 2 shows the correlation, colored by EDSS and separated between controls and pwMS, between pairs of intra- and interhemispheric SC measures. The strongest correlation was found when comparing the commissural ratio against the efficiency (*r* = −0.43 for pwMS, *r* = −0.41 for HC, *p* < 0.00001). Figure 3 shows the same comparison separated by center, also showing the same tendency for each separated center. All tests have been corrected by multiple comparisons.

**Figure 4.**
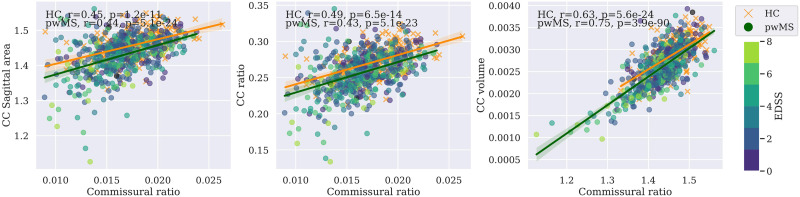
Correlation between interhemispheric measures. Pearson correlation between commissural ratio, a diffusion MRI-derived interhemispheric measure, and three T1 structural-derived interhemispheric measures (CC sagittal area (mm^2^), CC area ratio, and CC volume (mm^3^)). pwMS is colored by EDSS. HC is marked with a cross. Values are corrected by age and sex. Data are harmonized across centers using ComBat. Tests results are corrected for multiple comparisons. CC = corpus callosum; HC = healthy controls; pwMS = people with MS; EDSS = Expanded Disability Status Scale.

Figure 4 shows the relationship between the different measures of interhemispheric connectivity, corrected by age and sex and separated between pwMS and HC. Significant correlations were observed, with no major differences between HC and pwMS (*r* = 0.49 and 0.43 between commissural ratio and CC ratio for HC and pwMS, respectively, and *r* = 0.63 and 0.75 between commissural ratio and CC volume for HC and pwMS, respectively; *p* < 0.0001 in both cases).

Table 2 displays correlations—corrected by center, age, and sex—between structural measures, EDSS/SDMT, and the inter-/intraconnectivity measures. WMF was strongly associated with all connectivity values, especially with commissural ratio (*r* = 0.51, *p* < 0.0001) and efficiency (*p* = −0.46, *p* < 0.0001). LVF was also strongly associated with commissural ratio (*r* = −0.41, *p* < 0.0001). Higher cognitive scores were associated with increased connectivity, as shown by the correlation between the CC ratio and SDMT (*r* = 0.33, *p* < 0.0001). Intrahemispheric connectivity also displayed associations with EDSS (*r* = 0.18 with efficiency, *p* < 0.001) and SDMT (*r* = 0.24 with efficiency, *p* < 0.0001).

**Table 2.** Partial correlations with structural measures, EDSS and SDMT

	GMF	WMF	LVF	EDSS	SDMT
Commissural ratio	***r* = 0.23, *p* < 0.0001**	***r* = 0.51, *p* < 0.0001**	***r* = −0.41, *p* < 0.0001**	*r* = −0.16, *p* = 0.0082	***r* = 0.27, *p* < 0.0001**
CC ratio	*r* = −0.05, *p* = 1.0	***r* = 0.33, *p* < 0.0001**	***r* = −0.22, *p* < 0.0001**	***r* = −0.20, *p* < 0.0001**	***r* = 0.33, *p* < 0.0001**
Mean SPL	*r* = −0.00, *p* = 1.0	***r* = 0.39, *p* < 0.0001**	*r* = −0.14, *p* = 0.0057	*r* = −0.15, *p* = 0.03	***r* = 0.22, *p* < 0.0001**
Mean eff.	*r* = −0.06, *p* = 1.0	***r* = −0.46, *p* < 0.0001**	*r* = 0.15, *p* = 0.0023	*r* = 0.18, *p* = 0.0015	***r* = −0.24, *p* < 0.0001**
Commissural ratio/SC SPL	***r* = 0.26, *p* < 0.0001**	***r* = 0.40, *p* < 0.0001**	***r* = −0.40, *p* < 0.0001**	*r* = −0.12, *p* = 0.26	***r* = 0.22, *p* < 0.0001**
Commissural ratio/SC eff.	***r* = 0.20, *p* < 0.0001**	***r* = 0.54, *p* < 0.0001**	***r* = −0.38, *p* < 0.0001**	*r* = −0.18, *p* = 0.0012	***r* = 0.29, *p* < 0.0001**
CC ratio/SC SPL	*r* = −0.05, *p* = 1.0	*r* = 0.03, *p* = 1.0	*r* = −0.10, *p* = 0.18	*r* = −0.09, *p* = 1.0	*r* = 0.15, *p* = 0.0076
CC ratio/SC eff.	*r* = −0.01, *p* = 1.0	***r* = 0.47, *p* < 0.0001**	***r* = −0.23, *p* < 0.0001**	***r* = −0.22, *p* < 0.0001**	***r* = 0.36, *p* < 0.0001**

Partial correlations between intra- and interhemispheric measures in groups of patients against structural measures, EDSS and SDMT. Results are corrected by age and sex. Data are harmonized across centers using ComBat. Test results are corrected for multiple comparisons. GMF = gray matter fraction; WMF = white matter fraction; LVF = lesion volume fraction; EDSS = Expanded Disability Status Scale; SDMT = Symbol Digits Modalities Test; CC = corpus callosum; SPL = shortest path length; Eff = efficiency; SC = structural connectivity; Corrected by multiple comparisons. Partial correlations with *p* < 0.0001 are highlighted in **bold**.

**Figure 5.**
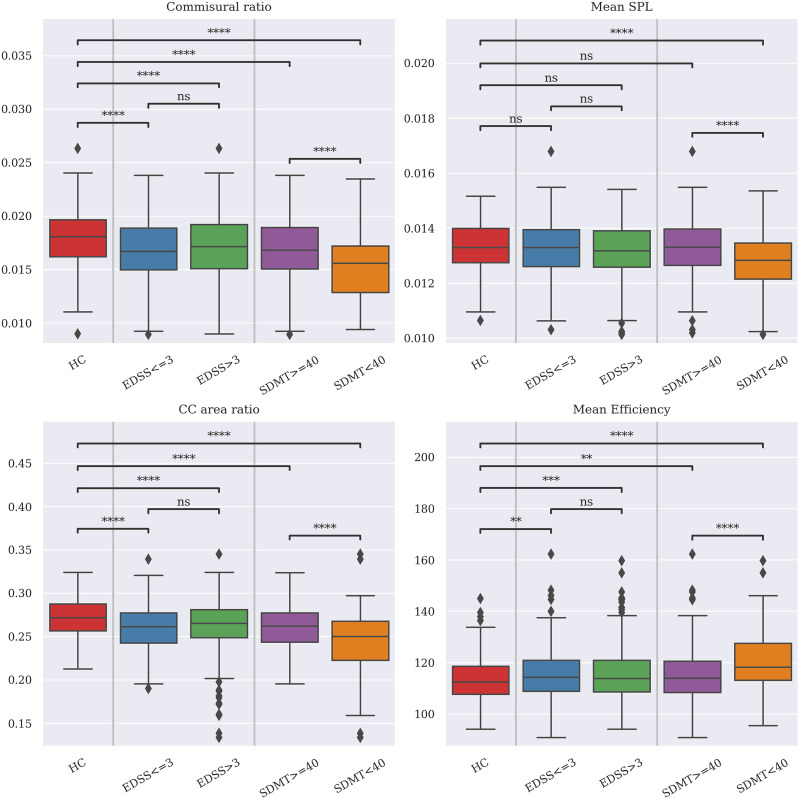
Comparison of connectivity values between HC and pwMS, divided in low/high EDSS and SDMT groups. Tests were conducted using two-way ANOVA analysis with three groups (HC and low/high EDSS and SDMT), and differences across groups were evaluated with post hoc pairwise Tukey HSD tests. Values are corrected by age and sex. Data are harmonized across centers using ComBat. Tests results are corrected for multiple comparisons. *N* = 697; *p < 0.05; **p < 0.01; ***p < 0.001; ****p < 0.0001. EDSS = Expanded Disability Status Scale; SDMT = Symbol Digit Modality Test; HC = healthy controls; pwMS = people with MS; SPL = shortest path length; CC = corpus callosum.

Figure 5 shows differences across high/low EDSS/SDMT for each of the connectivity values. Results showed that subjects with lower cognitive scores have a significantly lower intrahemispheric connectivity compared with both subjects with higher scores and HC. Furthermore, the differences between HC and pwMS in both groups were significant (*p* < 0.001) for SPL and efficiency.

Regarding the ratios between intra- and interhemispheric values, Table 2 displays correlations—corrected by center, age, and sex—between structural measures, EDSS and SDMT, and the calculated ratios. Using the commissural ratio, all the structural measures were strongly correlated to the inter/intra ratio, especially WMF (*r* = 0.54, *p* < 0.0001) and LVF (*r* = −0.38, *p* < 0.0001). SDMT was directly associated with the inter/intra ratio, especially when computed using the CC ratio and the efficiency (*r* = 0.33, *p* < 0.0001).

**Figure 6.**
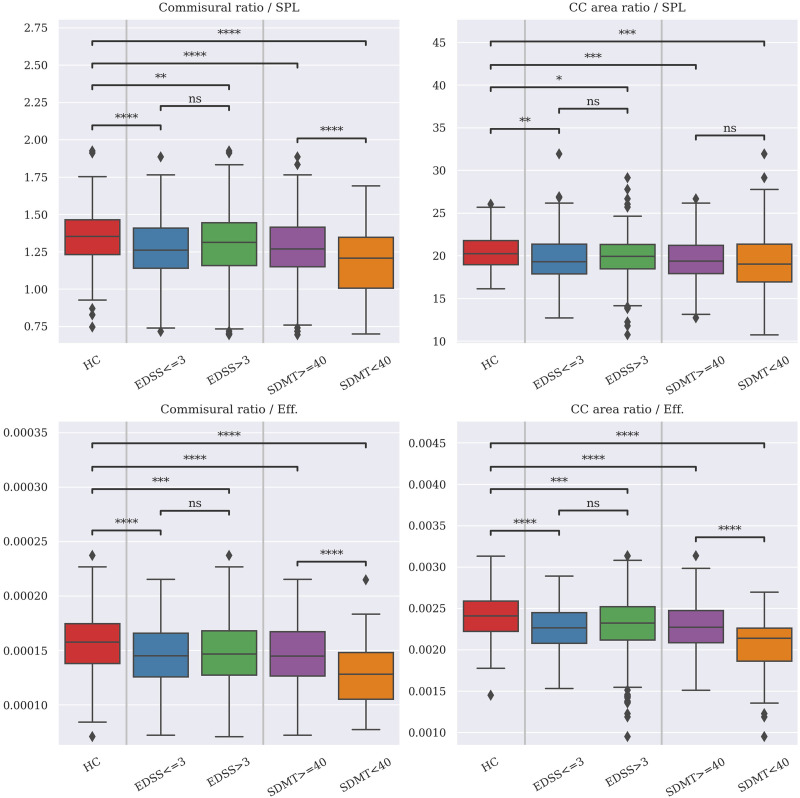
Comparison of inter/intra ratios between HC and pwMS, divided in low/high EDSS and SDMT groups. Tests were conducted using two-way ANOVA analysis with three groups (HC and low/high EDSS and SDMT), and differences across groups were evaluated with post hoc pairwise Tukey HSD tests. Values are corrected by age and sex. Data are harmonized across centers using ComBat. Tests results are corrected for multiple comparisons. *N* = 697; *p < 0.05; **p < 0.01; ***p < 0.001; ****p < 0.0001. EDSS = Expanded Disability Status Scale; SDMT = Symbol Digit Modality Test; HC = healthy controls; pwMS = people with MS; SPL = shortest path length; CC = corpus callosum; Eff. = efficiency.

Figure 6 shows differences across high/low values of EDSS/SDMT for each of the inter/intra ratios. When comparing between high/low values of EDSS/SDMT, differences between high and low SDMT (*p* < 0.0001) and HC versus the rest of the groups (*p* < 0.0001) are present in all cases, even after correcting for multiple comparisons.

## DISCUSSION

In this study, the conservation of brain connectivity across hemispheres in pwMS has been evaluated, and the relationship between such connectivity measures, brain structural measures, clinical disability, and cognition has been explored. Results suggest that the intra- and interhemispheric connectivity ratio is preserved in pwMS, as seen in HC or other mammals (Assaf et al., 2020; Krupnik et al., 2021). The ratio was preserved across all four combinations of intra- and interhemispheric measures, with the strongest correlation found when comparing the commissural ratio against the mean SPL. This supports the first part of our initial hypothesis, which was that the conservation principle would hold. The correlation was weaker when using the CC ratio as an alternative to interhemispheric measure. These results, combined with the stronger inter/intra ratio described above, seem to indicate that the commissural ratio, derived from fiber tracking, expresses different things: The CC ratio considers the interhemispheric connectivity sustained by the CC and only captures volumetric changes, while the commissural ratio includes all interhemispheric commissural systems.

Looking at specific relationships between disability and cognitive impairment and the intra- and interhemispheric connectivity, results showed that structural interhemispheric connectivity was significantly altered in MS, similar to Zhou et al. (2013). Moreover, strong correlations between WMF and LVF with interhemispheric connectivity were found, showing that lower WMF and higher LVF were associated with a diminished interhemispheric connectivity. These findings support our hypothesis that MS disrupts interhemispheric connectivity, with a lower CC ratio corresponding to higher EDSS and lower SDMT scores, as described by Ozturk et al. (2010). Supporting Information Figure S5 shows a visual representation of the discussed correlations. These findings support the idea that interhemispheric disconnection may play a more crucial role in the development of cognitive and disability deficits in MS (He et al., 2009).

Regarding intrahemispheric values, significant associations with WMF were found, suggesting a link between efficiency and WMF in the brain. Between high and low EDSS/SDMT groups, although significant differences were observed, particularly in efficiency, these differences were not as pronounced (no differences across EDSS groups, or between HC and high EDSS/low SDMT for the mean SPL). The studied metrics appear to be more sensible to cognitive decline captured by SDMT, rather than to physical disability captured by EDSS.

There was a strong link between WMF and LVF with inter/intra ratios, with higher WMF and lower LVF associated with a higher ratio, similar to the link found by He et al. (2009) between small-world network efficiency impairment and white matter lesion load. Higher SDMT and lower EDSS scores were associated with a higher ratio between connectivity values. This effect was more pronounced for SDMT, reinforcing the idea that that structural derived metrics are more highly related with cognition rather than with physical disability. This suggests that not only are individual inter-/intravalues related to structural damage, disability, and cognition, but the strength of the ratio also changes, either due to a decrease in interhemispheric connectivity or an increase in intrahemispheric connectivity. Considering the hypothesis that MS can alter interhemispheric connectivity (He et al., 2009), driven by the CC atrophy, a potential adaptative mechanism might be at play, in which intrahemispheric connectivity increases to maintain the balance between the two metrics. Indeed, if we divide the inter/intra ratio between subjects with high/low SDMT and EDSS values (Supporting Information Figure S6), we observe that the ratio survives for almost all groups and combinations. It is true that when using CC ratio as the interhemispheric measure, subjects with low EDSS show almost no correlation. However, as this does not happen with commissural ratio, this could also be the product of Simpson’s paradox after the division between groups, so further research should focus on replicating these results. To go further, longitudinal studies would be needed to prove a causal relationship confirming this mechanism. Moreover, it is possible that such interactions are affected by our specific dataset, analysis, or measures selected, highlighting the need for further validation to endorse this adaptative mechanism.

Our study presents several limitations that should be acknowledged. While Assaf et al. (2020) showed that the conservation principle holds using multiple scans, parameters, and even species, the multicentric dataset used in this work presents large differences across acquisition parameters (Supporting Information File S1): for example, on number of b-values in the diffusion, some centers having no bias field correction available, or variety of slice thickness. While we have applied ComBat harmonization to palliate this issue, a more controlled study design could eliminate this issue altogether and should be a focus of future works on this topic.

While this study highlights the importance of intra- and interhemispheric SC connectivity in MS, further investigation is required to determine whether these connectivity patterns are unique to MS or represent a more general feature of neurological disorders. We believe that the study of this mechanism could lead to a broader understanding on how MS affects people’s brains and how the brain tries to preserve its function. There is already a body of research on brain reorganization in MS (Schoonheim, Meijer, et al., 2015) (although mostly focusing on functional connectivity). Our findings add more evidence on this hypothesis. Moreover, other related dysregulations, such as interhemispheric imbalance (Iturria-Medina et al., 2011), warrant further exploration.

Functional connectivity was initially considered to be included in this work but was finally left out as we made the decision to focus on the SC. Nonetheless, other studies on functional connectivity have reported significant findings (Faivre et al., 2016; Jandric et al., 2022) for interhemispheric connections (Martínez et al., 2018) and homotopic connections in pwMS (Zhou et al., 2013). Thus, while this study is confined to the exploration of SC, future work could benefit from a multimodal approach that integrates both structural and functional connectivity metrics.

The results of this study suggest that the conservation of intra- and interhemispheric SC is preserved in pwMS. Lower ratios of inter/intraconnectivity are associated with atrophy, lesion load, cognitive impairment, and disability. These findings lend support to the hypothesis that while the conservation holds, interhemispheric connectivity decreases in more advanced stages of MS, while intrahemispheric connectivity increases to maintain the balance between the two. This has implications for neuroplasticity and brain resilience during the course of the disease. Longitudinal studies are needed to further explore this hypothesis and establish causal links.

## SUPPORTING INFORMATION

Supporting information for this article is available at https://doi.org/10.1162/netn_a_00404.

## AUTHOR CONTRIBUTIONS

Gerard Martí-Juan: Conceptualization; Data curation; Formal analysis; Investigation; Methodology; Software; Visualization; Writing – original draft. Jaume Sastre-Garriga: Conceptualization; Investigation; Resources; Supervision; Writing – review & editing. Angela Vidal-Jordana: Investigation; Methodology; Supervision; Writing – review & editing. Sara Llufriu: Data curation; Funding acquisition; Resources. Eloy Martínez-Heras: Data curation; Methodology; Software; Validation; Writing – review & editing. Sergiu Groppa: Data curation; Funding acquisition. Gabriel González-Escamilla: Data curation. Maria A. Rocca: Data curation; Funding acquisition. Massimo Filippi: Data curation; Funding acquisition. Einar A. Høgestøl: Data curation; Funding acquisition. Hanne F. Harbo: Data curation; Funding acquisition. Michael A. Foster: Data curation; Funding acquisition. Sara Collorone: Data curation. Ahmed T. Toosy: Data curation; Funding acquisition. Menno M. Schoonheim: Conceptualization; Funding acquisition; Investigation; Supervision. Eva Strijbis: Data curation. Giuseppe Pontillo: Data curation; Methodology. Maria Petracca: Data curation; Funding acquisition. Gustavo Deco: Conceptualization; Funding acquisition; Investigation; Methodology; Supervision; Validation; Writing – review & editing. Àlex Rovira: Data curation; Funding acquisition; Supervision. Deborah Pareto: Conceptualization; Funding acquisition; Investigation; Methodology; Project administration; Resources; Supervision; Validation; Writing – review & editing.

## COMPETING INTERESTS

G. Martí-Juan has received a MAGNIMS-ECTRIMS fellowship. J. Sastre-Garriga declares fees from Sanofi, Biogen, Celgene, Merck, Biopass, Novartis, and Roche and receives research support from Fondo de Investigación en Salud (PI19/00950) from Instituto de Salud Carlos III, Spain. A. Vidal-Jordana has received support for contracts from Juan Rodes (JR16/00024); receives research support from Fondo de Investigación en Salud (PI17/02162) from Instituto de Salud Carlos III, Spain; and has engaged in consulting and/or participated as a speaker in events organized by Novartis, Roche, Biogen, and Sanofi. S. Llufriu received compensation for consulting services and speaker honoraria from Biogen Idec, Novartis, TEVA, Genzyme, Sanofi and Merck. E. Martinez-Heras has nothing to disclose. S. Groppa has nothing to disclose. G. González-Escamilla has nothing to disclose. M. A. Rocca received speaker honoraria from Bayer, Biogen, Bristol Myers Squibb, Celgene, Genzyme, Merck Serono, Novartis, Roche, and Teva and receives research support from the MS Society of Canada and Fondazione Italiana Sclerosi Multipla. M. Filippi is the Editor in Chief of the Journal of Neurology and Associate Editor of Human Brain Mapping; received compensation for consulting services and/or speaking activities from Almiral, Alexion, Bayer, Biogen, Celgene, Eli Lilly, Genzyme, Merck-Serono, Novartis, Roche, Sanofi, Takeda, and Teva Pharmaceutical Industries; and receives research support from Biogen Idec, Merck-Serono, Novartis, Roche, Teva Pharmaceutical Industries, Italian Ministry of Health, Fondazione Italiana Sclerosi Multipla, and ARiSLA (Fondazione Italiana di Ricerca per la SLA). E. A. Høgestøl received honoraria for lecturing and for advisory board activity from Biogen, Merck, and Sanofi-Genzyme and has unrestricted research grant from Merck. H. F. Harbo has nothing to disclose. M. A. Foster is supported by an MRC grant (MR/S026088/1). S. Collorone is supported by the Rosetrees Trust (A1332, MS632), and she was awarded a MAGNIMS-ECTRIMS fellowship in 2016. A. Toosy has been supported by grants from MRC (MR/S026088/1), NIHR BRC (541/CAP/OC/818837), and RoseTrees Trust (A1332 and PGL21/ 10079); has had meeting expenses from Merck, Biomedia, and Biogen Idec; and was UK PI for two clinical trials sponsored by MEDDAY (MS-ON - NCT02220244 and MS-SPI2 - NCT02220244). M. M. Schoonheim serves on the editorial board of Neurology and Frontiers in Neurology; receives research support from the Dutch MS Research Foundation, Eurostars-EUREKA, ARSEP, Amsterdam Neuroscience, MAGNIMS, and ZonMW; and has served as a consultant for or received research support from Atara Biotherapeutics, Biogen, Celgene/Bristol Meyers Squibb, Genzyme, MedDay, and Merck. E. Strijbis has nothing to disclose. G. Pontillo has received research grants from ECTRIMS-MAGNIMS and ESNR. M. Petracca discloses travel/meeting expenses from Novartis, Roche, and Merck; speaking honoraria from HEALTH&LIFE S.r.l. and honoraria for consulting services from Biogen; and research grants from Baroni Foundation. A. Rovira serves/served on the scientific advisory boards for Novartis, Sanofi-Genzyme, Synthetic MR, TensorMedical, Roche, Biogen, and OLEA Medical; has received speaker honoraria from Bayer, Sanofi-Genzyme, Merck-Serono, Teva Pharmaceutical Industries Ltd., Novartis, Roche, Bristol-Myers, and Biogen; and receives research support from Fondo de Investigación en Salud (PI19/00950) from Instituto de Salud Carlos III, Spain. G. Deco has nothing to disclose. D. Pareto has received a research contract from Biogen Idec and receives research support from Fondo de Investigación en Salud (PI18/00823, PI22/01709) from Instituto de Salud Carlos III, Spain, co-funded by the European Union.

## FUNDING INFORMATION

Gerard Martí-Juan, European Committee for Treatment and Research in Multiple Sclerosis (https://dx.doi.org/10.13039/100008659), Award ID: ECTRIMS Research Fellowship Program 2021–2022.

## DATA AVAILABILITY STATEMENT

Data availability is restricted to data agreements between the Vall d’Hebron Research Institute and each participating center (https://github.com/GerardMJuan/GraphNetworkMarkers). MRI and the corresponding processed data are available upon request and data transfer approval with the corresponding center.

## Supplementary Material



## TECHNICAL TERMS

Structural connectivity: The physical wiring of the brain’s white matter pathways.
Corpus callosum: The largest white matter structure connecting the two cerebral hemispheres, enabling communication between the two.
Intra/interhemispheric connectivity: Connections within regions in one hemisphere and connections between the two hemispheres, respectively.
Homotopic connection: Connections between the corresponding regions in the left and right hemispheres.
Streamline: A representation of the path of a white matter fiber tract in tractography.
Anatomically constrained tractography: Method to perform fiber tracking in diffusion MRI using anatomical information from image segmentation to enhance accuracy.
Intersite variability: Differences in data across multiple research locations due to different acquisition machines, protocols, etc.
Principal components analysis (PCA): A dimensionality reduction technique by transforming variables into orthogonal components, facilitating visualization in lower dimensions of high dimensionality data.
Simpson’s paradox: A trend or correlation that appears in various groups of data, but disappears or reverses when analyzing the groups together.
