# Supplementary material for: Conservation of structural brain connectivity in people with multiple sclerosis
Source: Netw Neurosci. 2024 Dec 10;8(4):1545–62. doi: 10.1162/netn_a_00404 (PMC11674932; doi:10.1162/netn_a_00404)
Supplement: Supplementary file 1 [file netn-8-4-1545-s001.pdf]

Supplementary Material

May 27, 2024

1 Supplementary Data S1: Image acquisition parameters

For each center, with the same name as it is referred in the main text, we detail the image acquisition parameters provided by the participating centers for each of the sequences available that we have used in the paper.

1.1 Barcelona

- **Machine**: Siemens Magnetom Trio 3T with a 32-channel phased array coil.
- **Structural MRI**: 3T MP-RAGE sequence, TR=1800ms; TE=3.01ms; TI=900ms; 240 contiguous sagittal slices with voxel size = 0.94x0.94x0.94 mm3; matrix size=256 $\times$ 256.
- **FLAIR**: 3D FLAIR sequence, TR=5000ms, TE=397ms TI=1800ms; voxel size = 0.94$\times$0.94$\times$0,94 mm3; matrix size=256$\times$256.
- **DWI**: High Angular Resolution Diffusion Imaging (HARDI) sequence. TR=14800 ms; TE=103ms; voxel size=1.5x1.5x1.5 mm3; matrix size = 154$\times$154; b-value=1000 s/mm2 with 60 diffusion encoding directions and a single baseline image acquired at 0 s/mm2. Geometric distortions caused by field inhomogeneities were corrected through gradient field maps with the same slice prescription, slice thickness and field of view that DWI sequence.

- **Resting state fMRI**: TR=2000ms; TE=19ms; Voxel Size 1.7×1.7×3 mm3; Flip angle= 90º; Slices= 40; 450 volumes.

1.2 Mainz

- **Machine**: Siemens Magnetom Trio 3T with a 32-channel receive-only head coil.

- **Structural MRI**: 3T MP-RAGE sequence, TR=1900ms; TE=2.52ms; TI=900ms; voxel size = 1×1×1 mm3; flip angle = 9°; field of view = 256×256 mm2, matrix size = 256 ×256; slab thickness = 192 mm.

- **FLAIR**: 3D FLAIR sequence, TR=5000ms; TE=388ms; TI=1800ms; voxel size = 1×1×1 mm3; echo-train length=848; field of view = 256×256 mm2; matrix size = 256×256, slab thickness = 192mm.

- **DWI**: Single-shot echo-planar read-out, TR=9000ms; TE=102ms; voxel size=2×2×2.5 mm3; field of view=256x256 mm2, matrix size 128×128, b-value=0.900 s/mm2, 30 directions, single baseline image acquired at 0 s/mm2.

- **Resting state fMRI**: Gradient echo (GE)-EPI sequence, TR=3000ms; TE=30ms; voxel size = 3x3x3mm3; flip angle=90°, field of view =192×192mm^2; matrix size=64×64; slices=49; 200 volumes.

1.3 Milan

- **Machine**: Philips Ingenia CX 3T 32-channel head coil.

- **Structural MRI**: 3D T1 MP-RAGE sequence, TR=7ms; TE=3.2ms; TI=1000ms; voxel size = 1x1x1mm3; flip angle = 8º; field of view = 256x256mm2; matrix size = 256 x 256.

- **FLAIR**: 3D FLAIR sequence, TR=4800ms; TI=1650ms; voxel size = 1x1x1mm3; flip angle = 90º; echo-train length=167; field of view= 256x256mm2; matrix size = 256x256.

- **DWI**: Axial pulsed-gradient spin echo DW echo planar imaging, TR=5900ms, TE=78ms; voxel size =1.9x1.9x2.3mm3; flip angle=90º; FOV=240×233mm; matrix=112×85; b-value: 3 shells at b-value=700/1000/2855 s/mm

along 6/30/60 non-collinear directions and 10 b=0 volumes. Three additional b=0s/mm2 volumes with reversed polarity of gradients for distortion correction were acquired with a separate sequence.

- **Resting state fMRI**: Axial Gradient recalled resting state sequence, TR=1560ms; TE=35ms; voxel size = 2.5x2.5x3 mm3; echo train length = 47; flip angle = 70º; matrix size = 94x94; slices=48; 320 volumes.

1.4 Naples

- **Machine**: 3T Siemens Trio.
- **Structural MRI**: 3D T1 MPRAGE sequence, TR=3000ms; TE=2.41ms; TI=1000ms; voxel size = 0.8x0.8x0.8mm3; flip angle = 7º; field of view = 320x320mm2; matrix size=320x320.
- **FLAIR**: 3D FLAIR sequence, TR=6000ms; TE=404ms; TI=2200ms; voxel size = 1x1x1mm3; echo train length = 141; flip angle=120º; matrix size = 256x256.
- **DWI**: Echo planar imaging, TR=7400ms; TE=88ms, voxel size = 2.2x2.2x2.2mm3; flip angle = 90º; matrix size = 104x104; b value = 1000 s / mm2, 64 directions, with 7 additional images acquired at 0 s/mm2.
- **Resting state fMRI**: Echo planar imaging, TR=2500ms; TE=40ms; voxel size = 3x3x5mm3; flip angle = 90º; matrix size = 64x64; slices = 30; 200 volumes.

1.5 Oslo

- **Machine**: 3T General Electric DISCOVERY MR750 with 32 channel head coil.
- **Structural MRI**: 3D T1 BRAVO sequence, TR=8.16ms; TE=3.18ms; TI=450ms; voxel size = 1x1x1mm3; flip angle =12º; matrix size = 256x256;
- **FLAIR**: 3D T1 FLAIR sequence, TR=8000ms; TE=126.1ms; TI=2242ms; voxel size = 1.2x1x1mm3; flip angle = 90º; matrix size = 256x256.

- **DWI**: Echo planar imaging, TR=8150; TE=83.1ms; voxel size = 2x2x2mm3; flip angle = 90º; matrix size=128x128; b-value=1000 s/mm2 60 directions, with 5 additional images acquired at 0 s/mm2. Extra b=0s/mm2 volume with reversed polarity of gradients for distortion correction was acquired with a separate sequence: TR=8150; TE=83.1ms; voxel size = 2x2x2mm3; flip angle = 90º; matrix size=128x128.

- **Resting state fMRI**: Echo planar imaging resting state sequence, TR=2250ms; TE=30ms; voxel size = 2.7x2.7x3.5mm3; flip angle =79º; matrix size = 96x96; slices = 43; 200 volumes.

1.6 London

- **Machine**: 3T Philips Achieva.

- **Structural MRI**: 62 subjects with 3D T1 MP-RAGE, TR=6.82ms; TE=3.08ms; voxel size = 1x1x1mm3; echo train length = 230; flip angle = 8º; matrix size = 256x256. 29 subjects with 3D T1 MP-RAGE = TR=7ms; TE=3.2ms; voxel size = 1x1x1mm3; echo train length = 225; flip angle = 8º; matrix size = 256x256.

- **FLAIR**: 62 subjects with 3D FLAIR sequence, TR=8000ms; TE=387ms; TI=2400ms; voxel size = 1.2x1.1x1.1mm3; echo train length = 120; flip angle = 90º; matrix size = 224x224. 29 subjects with 3D FLAIR sequence, TR=5000ms; TE=3500ms; 1650ms; voxel size = 1x1x1mm3; echo train length = 177; flip angle = 90º; matrix size = 256x256.

- **DWI**: 62 subjects with spin echo dual coil echo planar imaging, TR=12000ms; TE=82ms; voxel size = 2.3x2.3x2.5mm3; echo train length = 47; flip angle = 90º; matrix size = 96x96; 3 shells with b-value = 300/711/2000 s/mm2 in 8/15/30 non-collinear directions and 7 b=0 volumes. An extra volume b = 0s / mm2 with reversed gradient polarity for distortion correction was acquired with a separate sequence: TR=6242ms; TE=96ms; voxel size = 2x2x2mm3; flip angle = 90º; matrix size=112x112;

- **Resting state fMRI**: Echo planar imaging sequence, TR=4000ms; TE=25ms; voxel size = 2.9x2.9x3.5mm3; echo train length = 27; flip angle = 90º; matrix size = 80x80; 43 slices; 200 volumes.

1.7 Amsterdam

- **Machine**: 3T General Electric Signa-HDxt.
- **Structural MRI**: 3D T1-weighted fast spoiled gradient echo sequence, TR=7.8ms; TE=3ms; TI=450ms; voxel size = 1.0x0.9x0.9 mm3; flip angle=12º.
- **FLAIR**: 3D FLAIR sequence, TR=8000ms; TE=125ms; TI=2350ms; voxel size = 1.2x0.98x0.98mm3.
- **DWI**: Echo planar imaging, TR=13000ms; TE=91ms, voxel size = 1x1x2.4 mm3; flip angle = 90º; b-value 1000 s/mm2 30 directions, with 5 images at b=0 s/mm2.
- **Resting state fMRI**: Echo planar imaging, TR=2200ms; TE=35ms; voxel size = 3.3x3.3x3mm3; flip angle = 80º; 46 slices; 202 volumes.

2 Supplementary Figure S2: Further cohort information

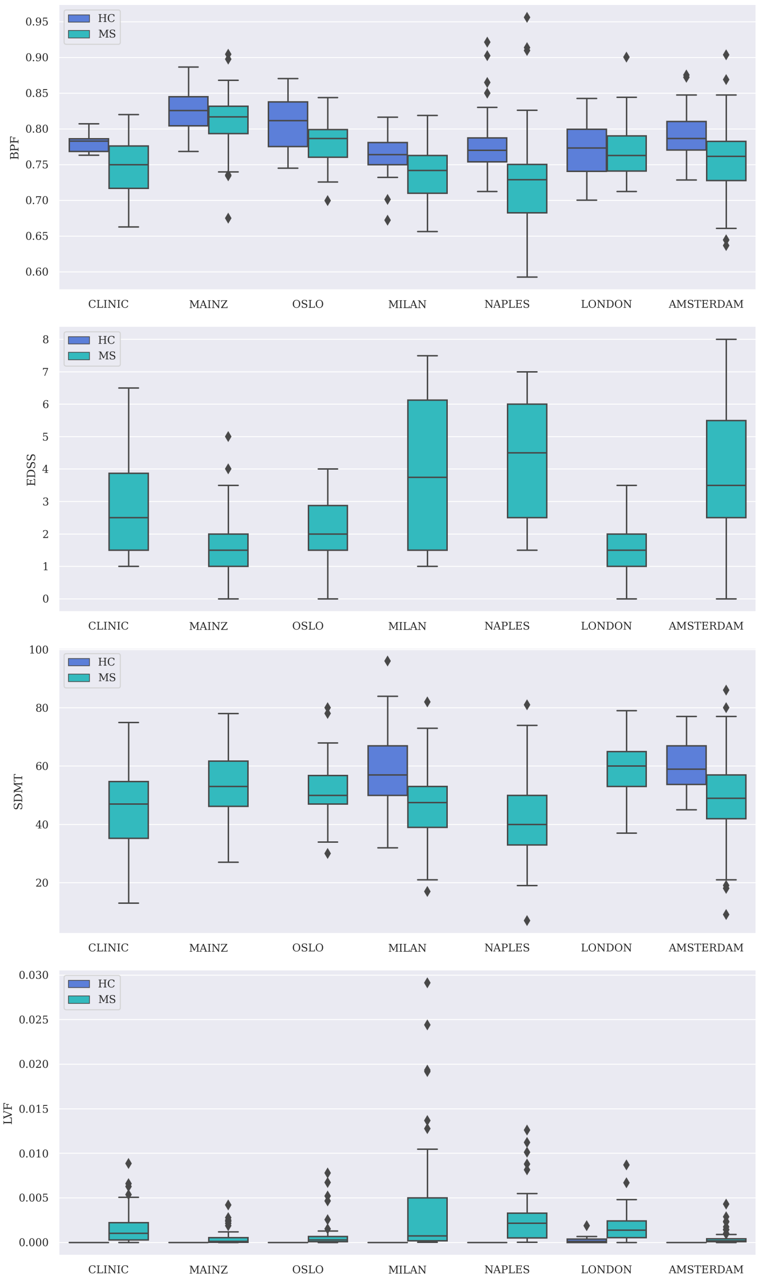

Comparison between cohorts and pwMS, HC for Brain Parenchymal Fraction, EDSS, SDMT and lesion volume fraction. pwMS: People with multiple sclerosis. HC: Healthy control. EDSS: Expanded Disability Status Scale. SDMT: Single Digit Modality Test

3 Supplementary Table S3: Association between years of education and SDMT

CENTER	t	pval
MILAN	0.902732	0.37
NAPLES	1.910250	0.06
OSLO	0.962597	0.34
LONDON	-0.288726	0.77
AMSTERDAM	1.592308	0.11

Table of the association using ordinary least squares regression between Years of Education and SDMT, for people with MS in the dataset, separated by center and corrected by age and sex. Centers where years of education were not available are not included. SDMT: Single Digit Modality Test. MS: Multiple Sclerosis

4 Supplementary Figure S4: Further comparison between and after ComBat harmonization

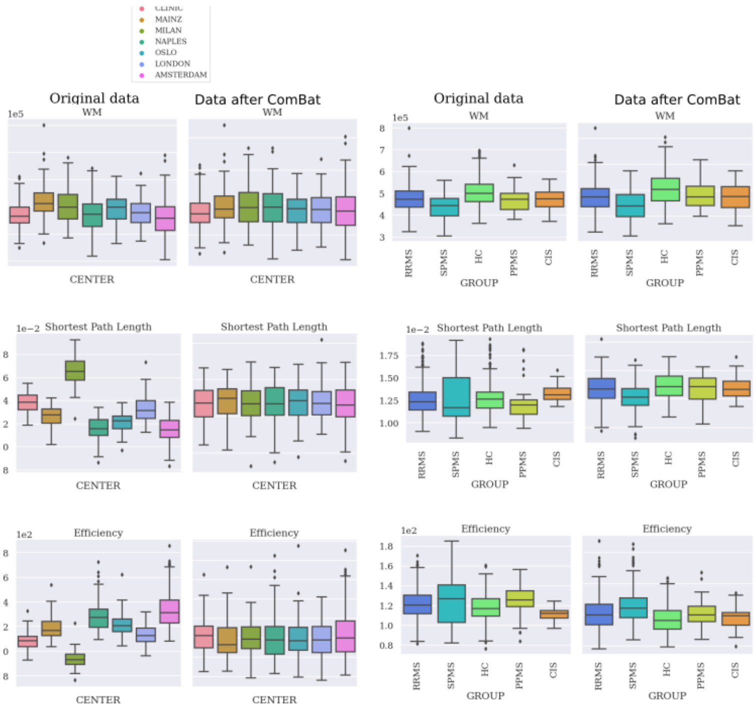

Comparison of the data before and after applying the ComBat harmonization procedure. Distribution separated by Centre (left) and group (right) before and after ComBat harmonization. WM: White Matter. RRMS: Relapsing-Remitting Multiple Sclerosis. SPMS: Secondary progressive MS. HC: Healthy Control. PPMS: Primary Progressive MS. CIS: Clinically Isolated Syndrome.

5 Supplementary Figure S5: Partial correlations with EDSS/SDMT

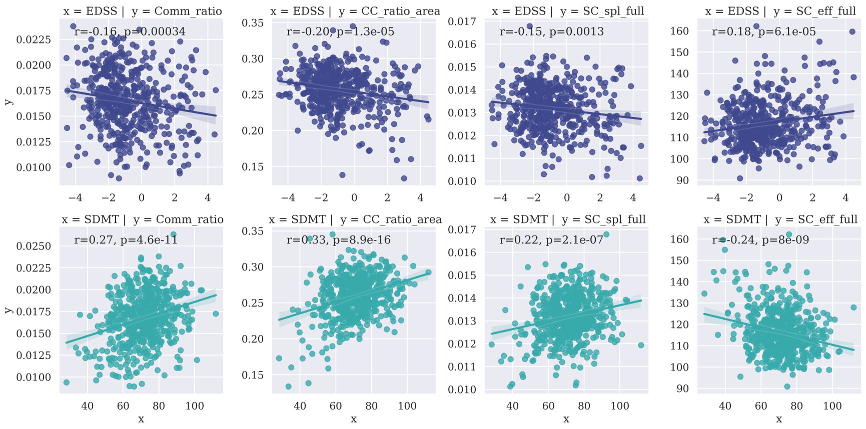

(a) Partial correlation between EDSS/SDMT with inter-intra connectivity values. Corrected by age, sex and center.

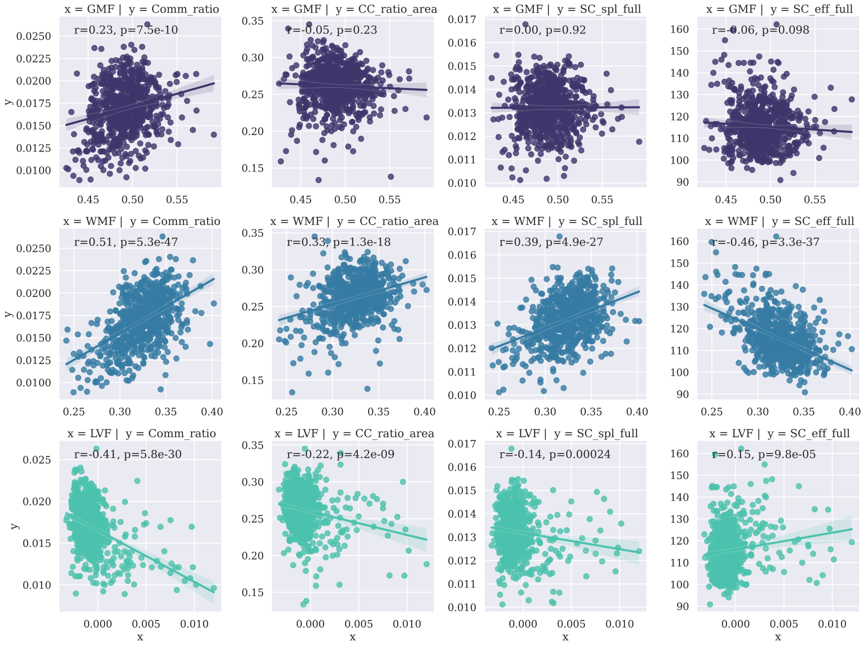

(b) Partial correlation between structural measures with inter-intra connectivity values. Corrected by age, sex and center.

Pearson correlations between EDSS and SDMT with inter-intra connectivity values. EDSS: Expanded Disability Status Scale. SDMT: Single Digit Modality Test. SC_spl_full_C: intrahemispheric Shortest path length. SC_eff_full_C: intrahemispheric Efficiency. Comm_ratio_C: Commisural fiber ratio. CC_ratio_area_C: Corpus callosum sagittal area ratio. GMF: Grey Matter Fraction. WMF: White Matter Fraction. LVF: Lesion Volume Fraction.

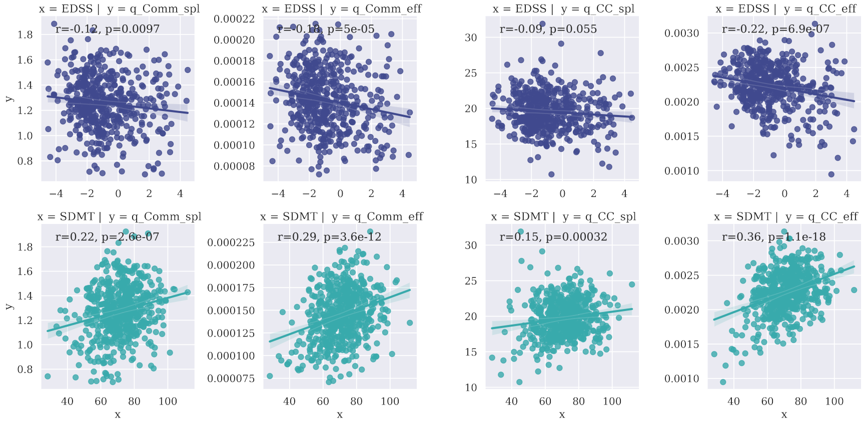

(c) Partial correlation between EDSS/SDMT with inter-intra ratios. Corrected by age, sex and center.

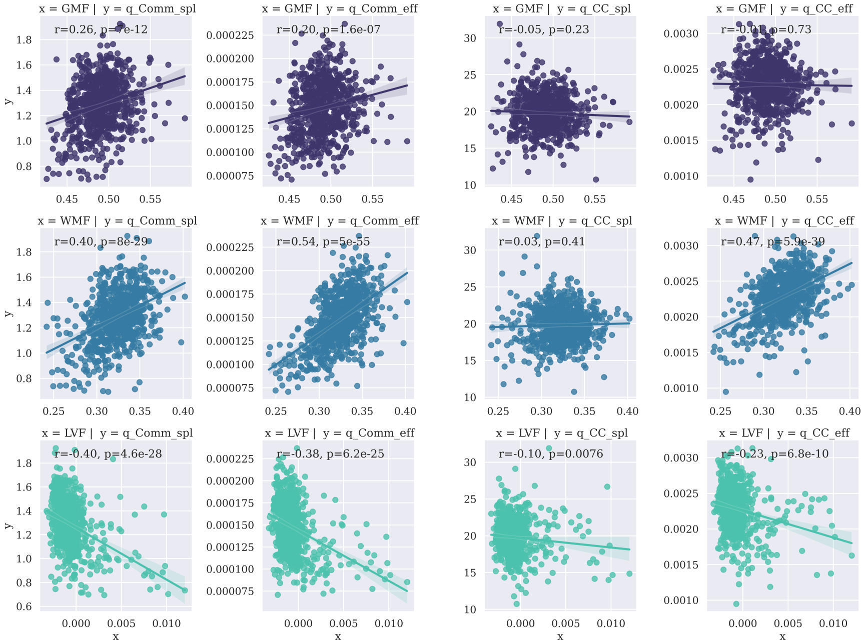

(d) Partial correlation between structural measures with inter-intra ratios. Corrected by age, sex and center.

Pearson correlation with inter-intra ratios. EDSS: Expanded Disability Status Scale. SDMT: Single Digit Modality Test. q_Comm_spl: Commisural ratio divided by Shortest path length. q_Comm_eff: Commisural ratio divided by Efficiency. q_CC_spl: Corpus callosum area ratio divided by shortest path length. q_CC_eff: Corpus callosum area ratio divided by efficiency. GMF: Grey Matter Fraction. WMF: White Matter Fraction. LVF: Lesion Volume Fraction.

6 Supplementary Figure S6: Intra-inter correlation separated by SDMT and EDSS

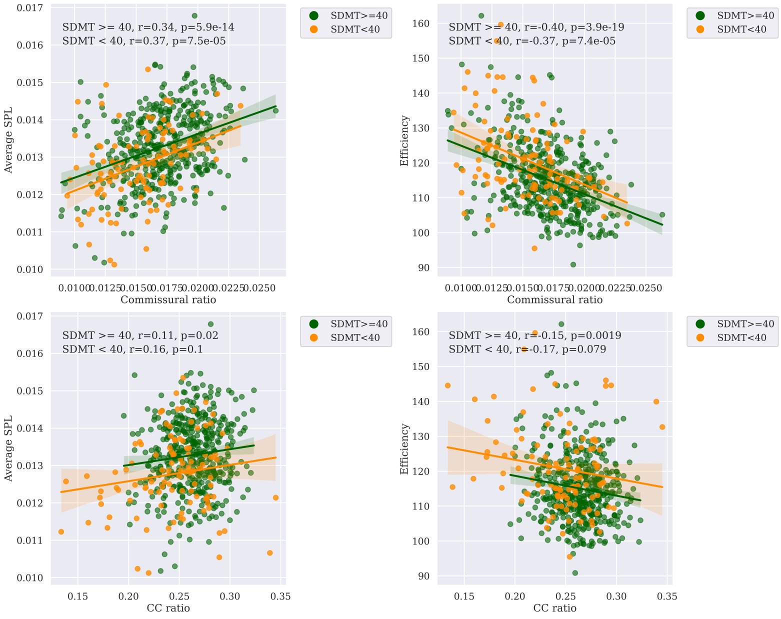

(e) SDMT groups

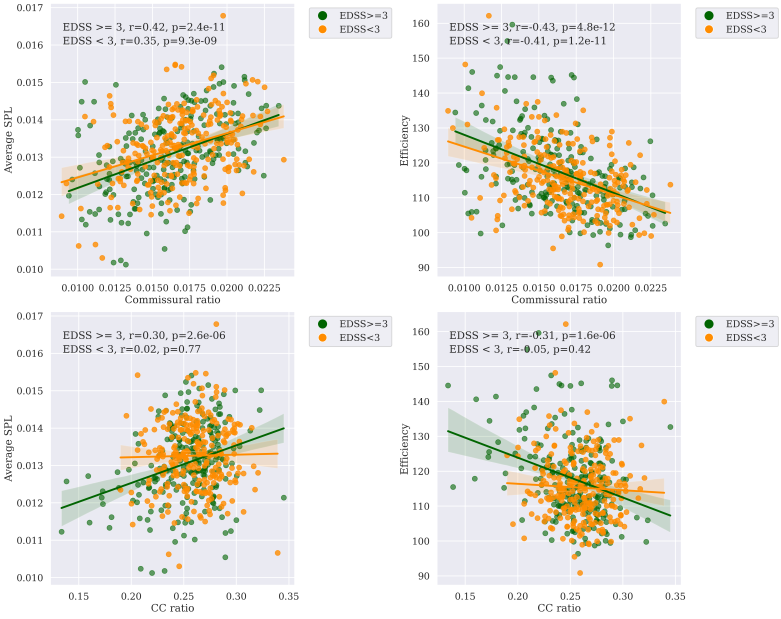

(f) EDSS groups

Each plot shows the Pearson correlation between the two variables, with the X axis representing the interhemispheric value and the Y axis representing the intrahemispheric value. Values are separated by high SDMT (>=40) and low (<40), and between EDSS high (>=3) and low (<3). Values corrected by age and sex. Data harmonized across centres using ComBat. SDMT: Single Digit Modality Test. HC: Healthy controls. pwMS: people with Multiple Sclerosis. SPL: Shortest path length. CC: corpus callosum.
